# Effect of a Tailored Physiotherapy Rehabilitation on Developmental Delay Primary to Non-communicating Hydrocephalus: A Case Study

**DOI:** 10.7759/cureus.61588

**Published:** 2024-06-03

**Authors:** Chaitali S Vikhe, H V Sharath, Raghumahanti Raghuveer, Swapnil U Ramteke

**Affiliations:** 1 Department of Sports Physiotherapy, Ravi Nair Physiotherapy College, Datta Meghe Institute of Higher Education and Research (Deemed to be University), Wardha, IND; 2 Department of Paediatric Physiotherapy, Ravi Nair Physiotherapy College, Datta Meghe Institute of Higher Education and Research (Deemed to be University), Wardha, IND; 3 Department of Neurophysiotherapy, Ravi Nair Physiotherapy College, Datta Meghe Institute of Higher Education and Research (Deemed to be University), Wardha, IND

**Keywords:** physiotherapy intervention, hippotherapy, neurodevelopmental techniques, developmental delay, non-communicating hydrocephalus

## Abstract

This case report presents the physiotherapy intervention of a one-year-old male child diagnosed with non-communicating hydrocephalus primary to developmental delay. Hydrocephalus is marked by an accumulation of cerebrospinal fluid and often leads to significant developmental delays and neurological impairments in affected infants. The physiotherapy intervention aimed to achieve head and trunk control, improve sensory awareness, and enhance overall body coordination and balance. Various techniques, including neurodevelopmental techniques, sensory stimulation, hippotherapy, and sensory integration therapy, were utilized to target specific developmental milestones and functional abilities. Outcome measures, including the Gross Motor Function Measure, Infant Neurological International Battery, Hammersmith Infant Neurological Examination, and New Ballard Score, were used to assess the patient's progress pre- and post-intervention. Significant improvements were observed across all outcome measures following four months of physiotherapy rehabilitation. The patient demonstrated substantial gains in gross motor function, neurological examination scores, and overall developmental milestones. These findings underscore the effectiveness of physiotherapy rehabilitation in addressing developmental delays associated with non-communicating hydrocephalus. This case underscores the significance of early physiotherapy intervention, which plays a vital role in enhancing outcomes and improving the quality of life for affected children.

## Introduction

Hydrocephalus is characterized by disturbances in the cerebrospinal fluid (CSF) circulation, resulting in imbalanced CSF flow dynamics, and can manifest as a symptom of an underlying disease [1]. It is characterized by an abnormal accumulation of CSF, which may cause alterations in intracranial pressure [2]. Occurring across all age groups, hydrocephalus stands as a significant cause of mortality and morbidity worldwide [3], with an estimated prevalence of 1.1 per 1,000 infants [4]. Non-communicating hydrocephalus occurs due to a blockage in the flow of CSF within the ventricular system [5], leading to increased pressure within the ventricles and subsequent hydrocephalus [6]. Despite its significant global impact, reliable data on the annual incidence of pediatric hydrocephalus, especially in low- and middle-income countries, are limited. Therefore, a coordinated global effort is urgently needed to address hydrocephalus, particularly in areas with high demand. This effort aims to reduce its incidence, morbidity, mortality, and disparities in access to treatment [7].

Evidence suggests that various molecular changes play a role in the development of hydrocephalus, with genetic factors possibly responsible for approximately 40%-50% of cases. Genetic predisposition, coupled with acquired risk factors, can accelerate ventricular enlargement. Primary hydrocephalus can also stem from developmental disorders linked to central nervous system birth defects, such as neural tube defects, arachnoid cysts, Dandy-Walker syndrome, and Chiari malformations [8,9].

Developmental delay is typically identified when a child fails to achieve developmental milestones at the anticipated times in comparison to their peers [10], indicating a delay in any dimension of functioning at the expected age [11]. Hydrocephalus often correlates with developmental delays and multiple comorbidities [12], often necessitating treatment or surgical intervention [13]. Understanding the interplay between hydrocephalus, developmental delay, and the role of physiotherapy is crucial for providing comprehensive care and improving outcomes for affected children.

## Case presentation

This is the case of a one-year-old male child. The mother gave a natal history of NICU (neonatal intensive care unit) admission for 15 days due to low birth weight. On December 5, 2023, the mother observed that the child was experiencing involuntary movements, so they took him to a private hospital, where he was admitted for three days. He was then referred to a neurosurgeon in Nagpur. Following a thorough investigation, the baby was diagnosed with hydrocephalus. After four days, he experienced the same episode and has been on medication since. At the age of one year, the mother noticed that the baby lacked neck control and was unable to sit or stand. Consequently, his parents decided to seek medical attention for the child at Acharya Vinoba Bhave Rural Hospital (AVBRH) in Wardha, India. Following thorough investigations, the doctors diagnosed him with a delay in developmental milestones and therefore referred him to physiotherapy. Upon assessment, it was determined that his developmental age was two months, whereas his chronological age was one year.

Clinical findings

Before the examination, the patient's informed consent and assent were obtained, after which a physical examination was conducted. However, developmental milestones were not achieved according to his developmental age. A detailed timeline of events is provided in Table 1.

**Table 1:** Timeline of events AVBRH: Acharya Vinoba Bhave Rural Hospital

Events	Timeline
Date of birth	06/02/2023
Admitted to a private hospital for low birth weight	06/02/2023
Got diagnosed with non-communicating hydrocephalus	05/12/2023
Visited AVBRH for the abovementioned complaints	03/01/2024
A physiotherapy assessment is performed	03/02/2024

Developmental milestones associated with gross motor skills were not achieved. A comprehensive summary of gross motor development is outlined in Table 2.

**Table 2:** Developmental milestones related to gross motor skills

Gross motor	Normal	Attained month
Head control	6 weeks	Partially attained
Rolling	4-6 months	Not attained
Sitting	5-7 months	Not attained
Creeping	6-8 months	Not attained
Crawling	9-11 months	Not attained
Standing with support	9-12 months	Not attained

Table 3 presents fine motor skills. The grasp reflex was acquired at seven months, but reaching, releasing, mouthing, transferring, and grasping were not attained within the anticipated timeframes.

**Table 3:** Developmental milestones associated with fine motor skills

Fine motor	Normal	Attained month
Grasp reflex	0-3 months	7 months
Reach	2-4 months	Not attained
Release	3-6 months	Not attained
Mouthing	3-6 months	Not attained
Transfer	4-5 months	Not attained
Grasp	6-8 months	Not attained

Table 4 displays language acquisition milestones. Turning head to sound was accomplished at eight weeks and cooing at six months, but monosyllables and disyllables were not achieved at six and nine months, respectively.

**Table 4:** Language acquisition milestones

Language	Normal	Attained month
Turns head to sound	6 weeks	8 months
Cooing	3 months	6 months
Monosyllables	6 months	Not attained
Disyllables	9 months	Not attained

Table 5 illustrates personal and social interaction milestones. Social smile was achieved at 11 months and recognizing the mother at seven months, but smiling at a mirror image and waving bye-bye were not attained at six and nine months, respectively.

**Table 5:** Developmental milestones associated with personal and social interactions

Personal and social	Normal	Attained month
Social smile	1 month	11 months
Recognizing mother	3 months	7 months
Smiles at the mirror image	6 months	Not attained
Waves bye-bye	9 months	Not attained
Personal and social	Normal	Attained month

Table 6 outlines primitive reflexes. The sucking reflex was present immediately after birth, the Moro reflex emerged at four to six months, but the crossed extension was absent at two months. Other reflexes were integrated within the anticipated timeframes.

**Table 6:** Various reflexes observed in infants and their typical developmental timeline

Reflexes	Normal	Present/integrated
Sucking reflexes	Immediately after birth	Present
Moro reflexes	4-6 months	Absent
Grasp reflexes	Immediately after birth	Present
Flexor withdrawal	2 months	Present
Extensor thrust	2 months	Absent
Crossed extension	2 months	Absent
Startle	3 months	Present

Physiotherapy intervention

In Table 7, a tailored rehabilitation protocol was implemented, spanning two months with daily hour-long sessions. Additionally, strategies for home-based activities were provided to the primary caregiver [14-16].

**Table 7:** Tailored physiotherapy intervention

Goals	Intervention	Procedure	Dosage
To achieve neck control	Neurodevelopmental techniques	Facilitation of neck-holding on a Swiss ball	6 days/week, 45 minutes per session
		Rolling on the flat surface	
		Performing neck extension on a Swiss ball while in a prone position, incorporating scapular retraction	
		Rolling with a downward weight shift	
		Stretching intercostal muscles, progressing to a pull-to-sit	
		Weight-bearing on one hand in a side-sitting position	
		Prone on elbows and prone on hands on a Swiss ball	
		Sitting on a tilt board and performing weight shifts	
		Sitting upright in a 90-90 position on a small stool	
Facilitate trunk control		Transitioning from supine to sitting, changing from standing to sitting, performing unilateral weight shifts while sitting, engaging in quadruped rocking and reaching activities, crawling, sliding with support, and enhancing trunk control on a Swiss ball	6 days/week, 45 minutes per session
		Reaching with arms while using standing frames	
		Applying perturbations to a child in a standing position	
To improve sensory awareness to relax and elongate the muscles	Sensory stimulation	Vestibular system stimulation: stimulating the vestibular system through head nods, head turns, swinging on a horizontal plane in a supine position, vertical plane cribbing, rolling on a Swiss ball, and bouncing on a Swiss ball in sitting	5 days/week for 5 min each activity per session
		Proprioceptive system stimulation: stimulating the proprioceptive system by tightly rolling into a blanket, applying joint compression to all extremity joints, using vibrations with a brush, using weighted blankets, and applying firm deep pressure by rolling a large ball over the child's body	
		Muscle relaxation/activation: the horse is kept at a consistent relaxed pace or a brisker pace, walking along straight paths and gentle curves	
To improve overall body awareness, coordination, and balance	Sensory integration therapy	Proprioception phase: patients engage in track walking or crawling, kicking a ball, participating in a wheelbarrow relay and doing sit-ups	5 days/week for 5 min each activity per session
		Vestibular phase: these activities stimulate the vestibular system and promote relaxation. Patients hammock, perform somersaults, pass objects over a rainbow-shaped structure, engage in scooter board tummy glides, and roll on various surfaces	
		Mixed phase: in this phase, activities focus on enhancing both proprioception and vestibular function. Patients participate in wall stepping, track walking or crawling, doing sit-ups for yoga, including various poses, wheelbarrow walking hammock, passing objects over a rainbow structure, and performing somersaults, twisting exercises, and rolling on different surfaces	

**Figure 1:**
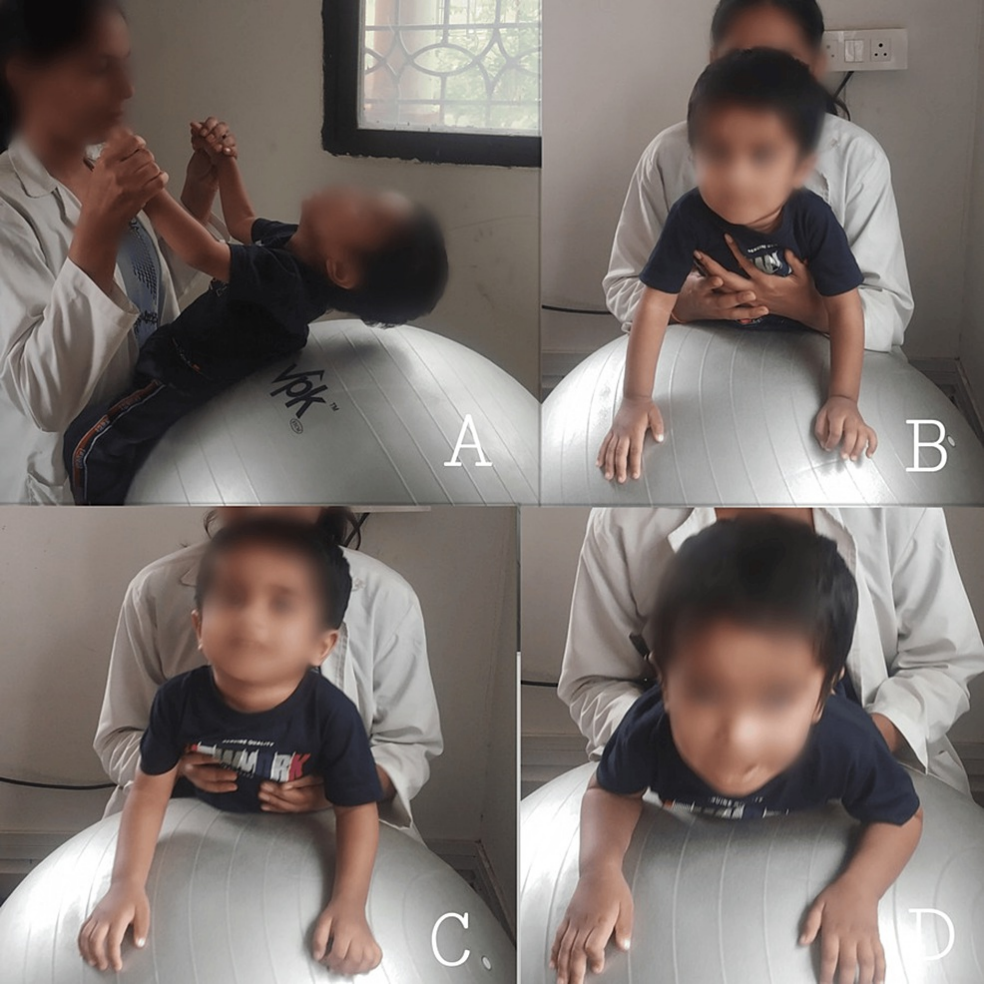
Neurodevelopmental techniques to improve neck control (A) Facilitation of neck-holding on a Swiss ball; (B) prone on elbows; (C) prone on hands; and (D) neck extension on a Swiss ball prone with scapular retraction

Figure 1 illustrates neurodevelopmental techniques aimed at enhancing neck control.

**Figure 2:**
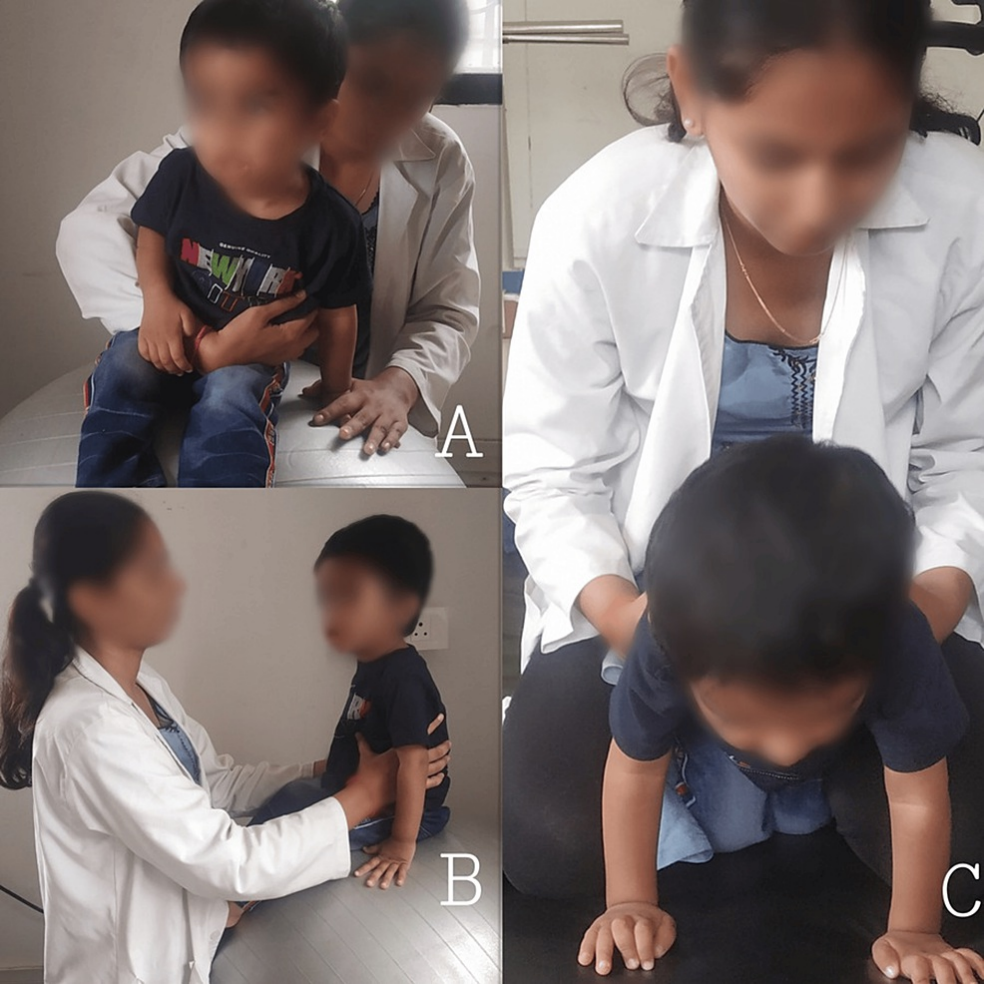
Neurodevelopmental techniques to facilitate trunk control (A) Unilateral weight shifts in sitting; (B) trunk control on Swiss ball; and (C) quadruped rocking and reaching activities

Figure 2 demonstrates neurodevelopmental techniques to facilitate trunk control.

**Figure 3:**
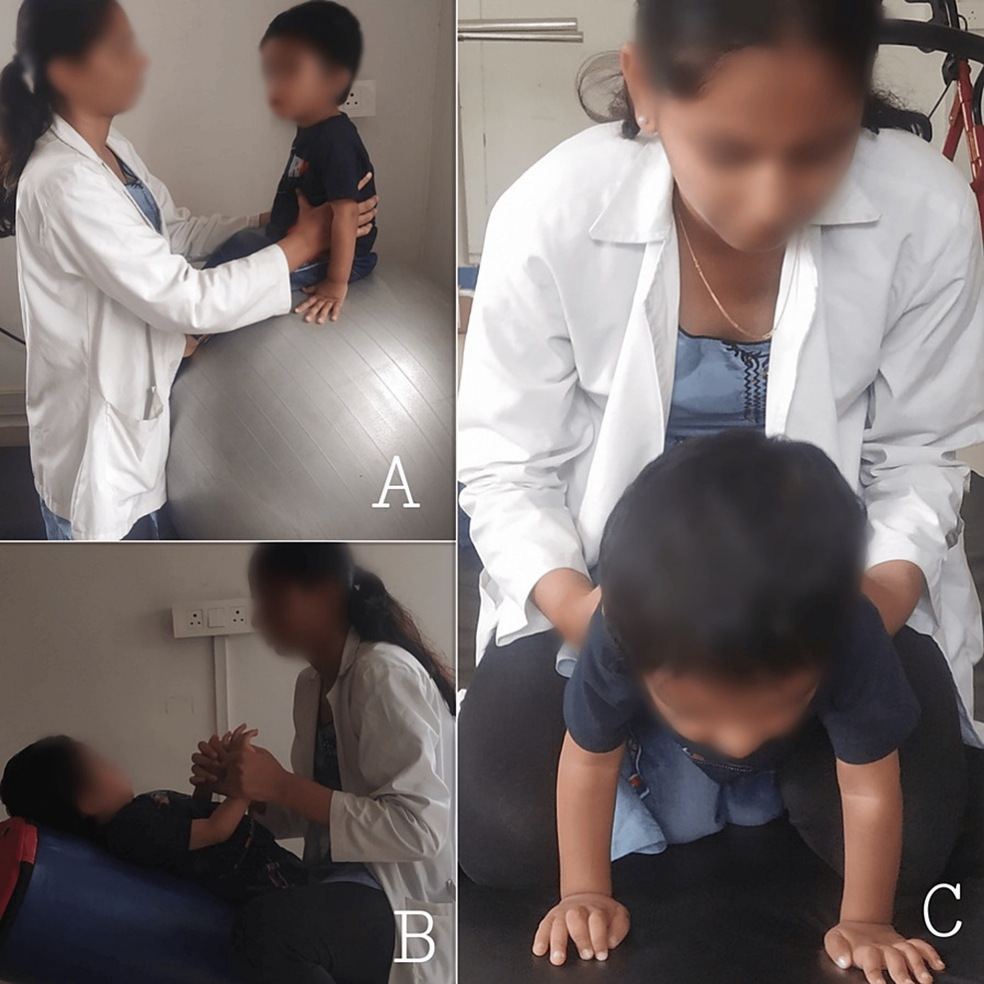
Sensory stimulation, including vestibular system stimulation and proprioceptive system stimulation, to improve sensory awareness (A) Bouncing on a Swiss ball; (B) swinging on the horizontal plane in the supine; and (C) joint compression focusing on all extremities

Figure 3 displays sensory stimulation methods, encompassing vestibular system stimulation and proprioceptive system stimulation, designed to improve sensory awareness.

Outcome measures

The study utilized various outcome measures to assess gross motor functions, neurological examination findings, and musculoskeletal system status both before (pre) and after (post) physiotherapeutic interventions. Table 8 presents the pre- and post-physiotherapy rehabilitation outcomes, demonstrating significant improvements across the evaluated parameters.

**Table 8:** The pre- and post-physiotherapy rehabilitation outcomes GMFM: Gross Motor Function Measure

Outcome measures	Pre-intervention	Post-intervention
GMFM	24%	45%
Hammersmith infant neurological examination	32	74
New Ballard Score	20	50

## Discussion

Hydrocephalus stands as one of the most prevalent neurosurgical diseases afflicting children globally [17]. While intraventricular hemorrhage associated with prematurity contributes to its occurrence, genetic changes associated with disease syndromes can also be causative factors [18]. Children diagnosed with hydrocephalus are at significantly increased risk of experiencing disabilities and delays in both mental and motor development, emphasizing the importance of continuous rehabilitation planning for pediatric cases [19]. This case report presents a physiotherapy rehabilitation approach for a one-year-old male child diagnosed with non-communicating hydrocephalus and developmental delay. The patient exhibited significant delays in achieving gross motor, fine motor, language, and personal/social developmental milestones compared to typical developmental timelines. These delays necessitated a multidisciplinary approach involving neurosurgery, pediatrics, and physiotherapy to address the underlying hydrocephalus and developmental delays.

Despite the conventional use of Bobath or neurodevelopmental therapy (NDT) in neurorehabilitation, evidence suggests the efficacy of alternative methods in improving motor function [20,21]. The physiotherapy intervention focused on achieving head control, facilitating trunk control, improving sensory awareness, and enhancing overall body awareness, coordination, and balance. Neurodevelopmental techniques, sensory stimulation, and sensory integration therapy were employed to address the specific needs of the patient. The intervention was administered six days a week for varying durations per session, tailored to the patient's developmental stage and tolerance.

Outcome measures, including the Gross Motor Function Measure, Infant Neurological International Battery, Hammersmith Infant Neurological Examination, and New Ballard Score, were used to assess the patient's progress pre- and post-intervention. Significant improvements were observed across all outcome measures after two months of physiotherapy rehabilitation. These findings underscore the effectiveness of physiotherapy rehabilitation in addressing developmental delays associated with non-communicating hydrocephalus. The physiotherapy rehabilitation program aimed to optimize motor development, functional abilities, and quality of life for pediatric patients with non-communicating hydrocephalus and developmental delays. It is essential to oversee certain limitations of the case report, such as the lack of long-term follow-up data and the lack of a group for comparison. Further research is warranted to explore the long-term effects of physiotherapy treatment in pediatric patients with hydrocephalus and developmental delays. Additionally, future studies should consider incorporating standardized outcome measures and larger sample sizes to provide more robust evidence of the effectiveness of physiotherapy rehabilitation in this population.

## Conclusions

This case report highlights the importance of physiotherapy intervention in managing developmental delays associated with non-communicating hydrocephalus. Physiotherapy rehabilitation plays a crucial role in optimizing outcomes and improving the quality of life for affected children, emphasizing the need for continued research and clinical innovation in this field.

## References

[1] Thomale UW (2021). Integrated understanding of hydrocephalus - a practical approach for a complex disease. Childs Nerv Syst.

[2] Karimy JK, Reeves BC, Damisah E (2020). Inflammation in acquired hydrocephalus: pathogenic mechanisms and therapeutic targets. Nat Rev Neurol.

[3] Tully HM, Dobyns WB (2014). Infantile hydrocephalus: a review of epidemiology, classification and causes. Eur J Med Genet.

[4] Maller VV, Gray RI (2016). Noncommunicating hydrocephalus. Semin Ultrasound CT MR.

[5] Gholampour S, Seddighi A, Fatouraee N (2024). Relationship between spinal fluid and cerebrospinal fluid as an index for assessment of non-communicating hydrocephalus. EBSCOhost.

[6] Figueiredo MV, Alexiou G, Laube KA, Manfroi G, Rehder R (2023). Novel concepts in the pathogenesis of hydrocephalus. Childs Nerv Syst.

[7] Li J, Zhang X, Guo J, Yu C, Yang J (2021). Molecular mechanisms and risk factors for the pathogenesis of hydrocephalus. Front Genet.

[8] Hochstetler A, Raskin J, Blazer-Yost BL (2022). Hydrocephalus: historical analysis and considerations for treatment. Eur J Med Res.

[9] Ha SY, Sung YH (2022). Abdominal and lower extremity muscles activity and thickness in typically developing children and children with developmental delay. J Exerc Rehabil.

[10] Gil JD, Ewerling F, Ferreira LZ, Barros AJ (2020). Early childhood suspected developmental delay in 63 low- and middle-income countries: large within- and between-country inequalities documented using national health surveys. J Glob Health.

[11] Mohamed M, Mediratta S, Chari A, da Costa CS, James G, Dawes W, Aquilina K (2021). Post-haemorrhagic hydrocephalus is associated with poorer surgical and neurodevelopmental sequelae than other causes of infant hydrocephalus. Childs Nerv Syst.

[12] Vikhe CS, Sharath HV, Brahmane NA, Ramteke SU (2024). The effect of physiotherapy intervention on an infant with congenital heart defect associated with developmental delay: a case report. Cureus.

[13] Chitlange NM, Sharath HV, Saklecha A, Desai S (2024). Effects of pediatric rehabilitation on children with spastic quadriplegia primary to seizure disorder and global developmental delay: a case report. Cureus.

[14] Sant N, Hotwani R, Palaskar P, Naqvi WM, Arora SP (2021). Effectiveness of early physiotherapy in an infant with a high risk of developmental delay. Cureus.

[15] Kraft KA, Weisberg J, Finch MD, Nickel A, Griffin KH, Barnes TL (2019). Hippotherapy in rehabilitation care for children with neurological impairments and developmental delays: a case series. Pediatr Phys Ther.

[16] Aukrust CG, Paulsen AH, Uche EO (2022). Aetiology and diagnostics of paediatric hydrocephalus across Africa: a systematic review and meta-analysis. Lancet Glob Health.

[17] Wallmeier J, Dallmayer M, Omran H (2022). The role of cilia for hydrocephalus formation. Am J Med Genet C Semin Med Genet.

[18] Sobana M, Halim D, Aviani JK, Gamayani U, Achmad TH (2021). Neurodevelopmental outcomes after ventriculoperitoneal shunt placement in children with non-infectious hydrocephalus: a meta-analysis. Childs Nerv Syst.

[19] Te Velde A, Morgan C, Finch-Edmondson M (2022). Neurodevelopmental therapy for cerebral palsy: a meta-analysis. Pediatrics.

[20] Koca TT, Ataseven H (2015). What is hippotherapy? The indications and effectiveness of hippotherapy. North Clin Istanb.

[21] Srushti Sudhir C, Sharath HV (2023). A brief overview of recent pediatric physical therapy practices and their importance. Cureus.

